# Surgical interventions for degenerative cervical disease: Impact on patient quality of life, mental health, pain relief, and spiritual health

**DOI:** 10.1016/j.heliyon.2024.e41555

**Published:** 2024-12-27

**Authors:** Ching-Ya Huang, Cheng-Shyuan Rau, Jo-Chien Lin, Shiun-Yuan Hsu, Ching-Hua Hsieh

**Affiliations:** a Department of Plastic Surgery, Kaohsiung Chang Gung Memorial Hospital and Chang Gung University College of Medicine, Kaohsiung, Taiwan, 83301; b Department of NeuroSurgery, Kaohsiung Chang Gung Memorial Hospital and Chang Gung University College of Medicine, Kaohsiung, Taiwan, 83301; c Department of Trauma Surgery, Kaohsiung Chang Gung Memorial Hospital and Chang Gung University College of Medicine, Kaohsiung, Taiwan, 83301

**Keywords:** Degenerative cervical disease, Surgery, Quality of life (QOL), Mental health, Pain relief, Spiritual health, Holistic well-being

## Abstract

**Introduction:**

Degenerative cervical diseases can severely affect patients' quality of life (QOL), mental health, and physical function. While surgical intervention is a common treatment, its impact on holistic well-being, including spiritual health, has not been thoroughly explored. This study aimed to evaluate the effects of surgery on QOL, pain-related disability, mental health, and spiritual well-being in patients with degenerative cervical diseases.

**Methods:**

This prospective cohort study was conducted with a purposive sampling of thirty-five patients who were diagnosed with degenerative cervical diseases and consented to surgery. The outcomes were assessed using the 36-item Short Form Health Survey Quality of Life Scale (SF-36), Patient Health Questionnaire-9 (PHQ9), Pain Disability Questionnaire (PDQ), and Holistic Well-being Scale (HWS) scales before surgery and six months postoperatively. The study focused on measuring the changes in patients’ physical and mental health, pain disability, and spiritual well-being, using paired sample t-tests.

**Results:**

Post-surgery, patients exhibited significant improvements in all domains of the SF-36 scores. Depression scores decreased as indicated by a reduction in PHQ-9 scores (from 18.54 to 15.00, P = 0.001). PDQ scores showed significant reductions in pain-related disability (from 79.00 to 50.26, P < 0.001). However, while there were improvements in emotional vulnerability (HWS score from 4.48 to 4.10, P = 0.020) and in the overall affliction score (from 4.45 to 4.11, P = 0.015), there were no significant changes in body irritability or spiritual disorientation within the affliction domain, nor in any of the items related to equanimity in holistic well-being.

**Conclusion:**

Surgical intervention for degenerative cervical diseases significantly improves patients’ QOL, mental health, and pain-related disability, but the impact on spiritual well-being is limited. This suggests that postoperative care should adopt a more holistic approach, integrating mental and spiritual support. Future research should include a more diverse sample, longer follow-up periods, and a combination of subjective and objective measures for a comprehensive assessment of outcomes.

## Lists of abbreviations

ACDF: Anterior discectomy with fusion
DCM: Degenerative cervical myelopathy
FSC: Functional Status Component
HWS: Holistic Well‐being Scale
QOL: quality of life
MCS: Mental Component Scale
mJOA: modified Japanese Orthopedic Association
PC: Psychosocial Component
PCS: Physical Component Scale
PDQ: The Pain Disability Questionnaire
PHQ9: Patient Health Questionnaire-9
SF-12: 12-item Short Form Survey
SF-36: 36-item Short Form Health Survey Quality of Life Scale

## 1 Introduction

Degenerative cervical diseases, encompassing conditions such as herniated discs, spinal stenosis, and foraminal stenosis, often lead to cervical radiculopathy and myelopathy, resulting in significant functional neurological decline, and disability. In addition, pain was significantly impacting the lower self-efficacy of the patients living with degenerative cervical myelopathy (DCM). Large areas of the body were common with pain, not just those traditionally associated with DCM [1]. It can also significantly affect mental health, contributing to conditions such as depression and anxiety. One of the most prevalent mental effects of degenerative cervical disease is the high incidence of depression and anxiety among patients. In a study by Sang et al. [2], approximately one-third of patients with cervical degenerative disc disease reported experiencing depression or anxiety. This is a concerning statistic given the already debilitating nature of the physical symptoms associated with the disease. The mental strain of dealing with chronic pain, disability, and the uncertainty surrounding treatment outcomes significantly affects these patients' quality of life (QOL) [[3], [4], [5]].

QOL is a subjective and multidimensional concept that reflects an individual's perception of their well-being, influenced by physical, mental, social, and cultural factors [6]. Defined as one's position in life relative to their goals and expectations, QOL varies greatly across individuals and contexts [7,8]. Research often uses patient-reported outcomes to assess QOL, though there is variability in how it is defined and measured, leading to inconsistencies in studies [7]. For example, studies frequently use various patient-reported outcome measures, such as the SF-36, EQ-5D, and PROMIS, which assess different QOL aspects depending on the dimensions and items included [7]. This variability in instruments often results in inconsistent findings, as QOL can be conceptualized as either an aggregate of specific issues, a single global item, or a label for multiple outcomes. In this study, we adopt the World Health Organization's definition of QOL as a person's perception of their position in life in the context of the culture and value systems in which they live, influenced by their goals, expectations, standards, and concerns [6]. Additionally, we use the 36-item Short Form Health Survey Quality of Life Scale (SF-36) to measure QOL across multiple dimensions, providing a standardized and validated assessment of both physical and mental health domains [9]. Recent research has shown that surgical treatment of degenerative cervical disease can lead to improvements in QOL [10,11,12,13]. Surgical interventions for degenerative cervical disease, including Anterior Cervical Discectomy and Fusion (ACDF), Posterior Cervical Decompression and Fusion (PCDF), decompression surgery, and hybrid approaches such as Cervical Hybrid Surgery (HS), have shown significant improvements in both physical, QOL and mental health outcomes [[10], [11], [14], [15], [16], [17]]. ACDF and PCDF are frequently used, with ACDF often associated with greater improvements in the Neck Disability Index and pain relief, while decompression surgery improves both functional and mental health by relieving spinal cord pressure [18,19]. Hybrid surgeries, combining ACDF and Cervical Disc Arthroplasty, also demonstrate enhanced pain reduction and QOL, particularly in multilevel degenerative cases [20]. However, pre-existing mental health conditions, such as depression and anxiety, may limit improvements, highlighting the need for individualized care [21]. Tetreault et al. found that patients with conditions like depression or bipolar disorder showed smaller gains in QOL post-surgery, emphasizing the importance of addressing mental health for optimal recovery in DCM [22].

In addition to the mental burden, patients often confront spiritual distress when facing chronic and degenerative illnesses. Spirituality can play a key role in how patients make sense of their illness, cope with suffering, and find meaning in their experience [23]. Spiritual health becomes a critical aspect of managing the disease, as many patients turn to their spiritual beliefs for comfort and guidance during these challenging times. While many studies have shown that surgical treatments for degenerative cervical diseases relieve physical symptoms and significantly improve holistic well-being—including mental health, functional abilities, and overall life satisfaction—research on the impact of these interventions on spiritual health remains limited [1,[10], [11], [14], [15],^19^,^24^,^25^]. In this study, we adopt the framework by Chan et al. [26], which defines spiritual health as comprising two core components: affliction, or spiritual and emotional distress, and equanimity, which reflects resilience and mindful acceptance. By examining these aspects, we aim to address the limited existing research on spiritual health in surgical interventions, exploring how it intersects with physical and mental health outcomes in patients with degenerative cervical disease. In summary, we hypothesize the following outcomes for patients undergoing surgery for degenerative cervical disease. First, we expect to observe improvements in QOL across physical and mental domains following surgery. Second, we anticipate reductions in pain-related disability and depressive symptoms. Finally, while we anticipate measurable improvements in physical and mental health, we also expect some degree of improvement in spiritual health. Together, these hypotheses guide our analysis and reflect the potential impact of surgical interventions across multiple dimensions of patient health.

## 2 Methods

### 2.1 Study design

This prospective cohort study was conducted at Kaohsiung Chang Gung Memorial Hospital, Taiwan, from July 2022 to June 2024. It employed purposive sampling and integrated questionnaire surveys with medical record collection to investigate patients’ QOL, depression score, pain disability, and spiritual health at two intervals: before surgery and six months after surgery among patients undergoing spine surgery at our institution. Before study commencement, ethical approval with the protocol number 202200381B0 was obtained from the Institutional Review Board of the hospital, and patient consent was duly secured. The collected medical record of the patient profile included sex, age, weight, height, occupation, marital status, education level, religious beliefs, comorbidities, smoke, alcohol consumption, betel nut use, primary companion(s) in the hospital, and primary companion(s) after discharge.

### 2.2 Study group selection

The inclusion criteria consisted of patients over the age of 20 diagnosed with degenerative cervical diseases, including intervertebral disc herniation, ossification of the posterior longitudinal ligament, and spinal stenosis, leading to cervical radiculopathy or DCM, who were scheduled to undergo ACDF surgery. All surgeries were performed by the same team (led by C.S.-R) to ensure consistency. Patients were required to be awake, able to communicate verbally, and provide informed consent after receiving full details of the study's objectives and risks. Before surgery, baseline data were collected, including demographic details, comorbidities, and lifestyle. Post-surgery, patients were monitored according to standard protocols, with follow-up assessments conducted six months later to evaluate changes in their health outcomes. Exclusion criteria included patients unable to attend follow-up visits, complete questionnaires, or provide reliable responses, as well as those with severe cognitive impairments.

### 2.3 The 36-item Short Form Health Survey Quality of Life Scale (SF-36), Taiwan version

The 36-item Short Form Health Survey Quality of Life Scale (SF-36) used in this study was developed by Ware in 1990 [9] as a multidimensional health-related QOL questionnaire. It is a general psychological measurement tool not specifically designed for any particular age group, disease, or treatment. It primarily measures respondents' perceived health status across eight dimensions, comprising a total of 36 items. The dimensions measured in this study are as follows: physical functioning (10 items), role limitations due to physical health problems (4 items), bodily pain (2 items), general health perceptions (5 items), vitality (4 items), social functioning (2 items), role limitations due to emotional problems (3 items), and mental health (5 items). Additionally, there is one item that assesses self-rated health changes. The Taiwan version (Short Form-36 Taiwan version) was translated and authorized for academic use by domestic scholars following the International Quality of Life Assessment Project on behalf of the original developer [9,27]. Additionally, we can aggregate the scores of the eight subscales into two composite scores: the Physical Component Scale (PCS) and the Mental Component Scale (MCS). PCS reflects physical functioning, role limitations due to physical health problems, bodily pain, and general health perceptions; while MCS reflects vitality, social functioning, role limitations due to emotional problems, and mental health. This approach allows for differentiation between physiological and mental aspects of health. The scoring ranges from 0 to 100, where 0 indicates the poorest health condition and 100 represents the best health condition [27].

### 2.4 Patient Health Questionnaire-9 (PHQ9)

The Patient Health Questionnaire-9 (PHQ9) is a widely used tool for assessing the severity of depression. This scale includes nine questions that align with the diagnostic criteria for depressive disorders in the Diagnostic and Statistical Manual of Mental Disorders (DSM)-IV. Each item on the PHQ-9 is rated on a scale from 0 (not at all) to 3 (nearly every day), allowing individuals to indicate how often they have been bothered by each symptom over the past two weeks [28]. Scores on the PHQ-9 can range from 0 to 27, with different thresholds indicating varying levels of depression severity. Scores 1–4 indicate minor depression, 5–9 mild depression, 10–14 moderate depression, 15–19 moderately severe depression, and 20–27 severe depression. Since its creation, researchers have established the validity and reliability of the PHQ-9 in a variety of settings [[29], [30], [31], [32]].

### 2.5 The Pain Disability Questionnaire (PDQ)

The Pain Disability Questionnaire (PDQ) is a tested tool designed to evaluate functional disability in individuals with chronic musculoskeletal disorders, offering a comprehensive score range from 0 to 150, with a higher score typically indicating a greater level of disability or functional impairment due to pain. The PDQ has been extensively validated and is recognized for its high reliability and internal consistency, with test-retest reliability coefficients ranging from 0.94 to 0.98 and a Cronbach's alpha of 0.96. Both core components of PDC, a functional status component (FSC) and a psychosocial component (PC), had been validated for their intended assessments following a factor analysis, with the minimal clinically important difference of PDQ being 20 [33].

### 2.6 Holistic Well‐being scale (HWS)

The 30-item Holistic Well-Being Scale (HWS) assesses spiritual resilience, which is defined as the absence of affliction and the presence of equanimity [26]. The HWS examines spiritual well-being with a focus on the dual aims of easing suffering and achieving lasting enjoyment by combining insights from Western psychology with Eastern traditions related to Confucianism, Buddhism, and Daoism. The perceived affliction, including emotional vulnerability, bodily irritability, and spiritual disorientation, as well as the perceived equanimity, defined by non-attachment, mindful awareness, general vitality, and spiritual self-care, were measured in the HWS. These perceived afflictions and equanimity comprise the concept of spiritual resilience. A ″0 to 10″ scale is used to rate each item; a score of 0 indicates complete disagreement, and a score of 10 indicates complete agreement. Whereas a high perceived equanimity score denotes good spiritual resilience, a high perceived affliction level denotes low spiritual resilience. The psychometric qualities of the HWS have been validated in a Hong Kong-based study of the Chinese population [26].

### 2.7 Statistical analysis

The study sample number is based on the number of samples required to calculate the *t*-test measurement of matched pairs. Using G-Power 3.1.9.2, setting the effect size (dz = 0.5) and the alpha error significant level to 0.05, the statistical level can be reached with a statistical 1-beta error of 0.90 and a sample requirement of 36. The encoded and archived data collected from legitimate questions are then analyzed using the IBM SPSS version 23 software package for Windows. When dealing with continuous variables, Levene's test is used first to assess variance homogeneity. Following that, the paired *t*-test and student's *t*-test are used to investigate differences between continuous variables. Continuous variables are presented as mean ± standard deviation or median ± interquartile range. Values for category variables are represented as percentages. A P-value of less than 0.05 indicates statistical significance.

## 3 Results

### 3.1 Patients’ demographics

Finally, 35 patients participated in this investigation by completing the entire survey. As shown in Table 1, the patient group consisted of a nearly even split between males (48.6 %) and females (51.4 %) with a wide age range, predominantly in the 40–49 age range (37.1 %). The patients' occupational backgrounds, marital statuses, and education levels varied, with the majority having married (48.6 %) and at least a senior high school education. Most patients identified with Buddhism (42.9 %) and had various comorbidities with hypertension the most (37.1 %), though a large portion did not engage in smoking, alcohol, or betel nut use. The primary companion(s) in the hospital were mainly spouses or cohabitants (42.9 %), followed by children (25.7 %). The primary companion(s) after discharge were mainly spouses or cohabitants (51.4 %), followed by children (34.3 %).

**Table 1:** Patients’ demographics.

Characteristics		N (%)/Mean ± SD
Total		35
Sex	Male	17 (48.6)
	Female	18 (51.4)
Height, cm		166.1 ± 8.7
Weight, kg		72.0 ± 14.0
Age range, years	30–39	3 (8.6)
	40–49	13 (37.1)
	50–59	10 (28.6)
	60–69	7 (20.0)
	70–79	2 (5.7)
Occupation	None	9 (25.7)
	Household	4 (11.4)
	Labor	6 (17.1)
	Business	6 (17.1)
	Governmental	2 (5.7)
	Other	8 (22.9)
Marital status	Single	6 (17.1)
	Married	17 (48.6)
	Divorced	6 (17.1)
	Cohabitation	1 (2.9)
	Widowed	5 (14.3)
Education level	Elementary	4 (11.4)
	Junior high	1 (2.9)
	Senior high	11 (31.4)
	Junior college	8 (22.9)
	University	7 (20.0)
	Graduate school	4 (11.4)
Religious beliefs	None	8 (22.9)
	Buddhism	15 (42.9)
	Taoism	9 (25.7)
	Christianity	3 (8.6)
Comorbidities	No	15 (42.9)
	Diabetes mellitus	6 (17.1)
	Hypertension	13 (37.1)
	Stroke	1 (2.9)
	CAD	4 (11.4)
	Hyperlipidemia	5 (14.3)
	Hepatitis B/C	1 (2.9)
	Other	4 (11.4)
Smoke	No	31 (88.6)
	Yes	4 (11.4)
Alcohol	No	31 (88.6)
	Yes	4 (11.4)
Betel nut	No	35 (100)
	Yes	0 (0.0)
Primary companion(s) in hospital	Spouse or cohabitant	15 (42.9)
	Parents	5 (14.3)
	Siblings	5 (14.3)
	Children	9 (25.7)
	Neighbors	1 (2.9)
	Friends	1 (2.9)
Primary companion(s) after discharge	Spouse or cohabitant	18 (51.4)
	Parents	9 (25.7)
	Siblings	3 (8.6)
	Children	12 (34.3)
	Neighbors	1 (2.9)
	Friends	1 (2.9)
	Other	1 (2.9)

### 3.2 Analysis of SF-36, PHQ9, and PDQ score before and after surgery (Table 2)

In terms of SF-36, PHQ9, and PDQ scores (Table 2), patients' physical and mental health improved significantly after 6 months postoperatively compared to previously. Specifically, the SF-36 scores increased significantly across all domains: physical functioning from 57.57 to 71.50 (P = 0.003), role-physical from 7.85 to 50.00 (P < 0.001), bodily pain from 43.11 to 61.90 (P < 0.001), general health improved from 46.37 to 57.86 (P = 0.011), and PCS from 38.72 to 60.31 (P < 0.001), indicating enhanced physical health and reduced pain. Similarly, vitality improved from 42.85 to 52.50 (P < 0.001), social functioning from 48.92 to 64.16 (P < 0.001), role-emotional from 31.42 to 58.88 (P = 0.010), mental health from 51.54 to 60.53 (P < 0.001), and MCS from 43.68 to 59.02 (P < 0.001), reflecting better overall and mental health post-treatment. The PHQ9 score improved from 18.54 to 15.00 (P = 0.001), indicating a decreased depression level. The FSC of PDQ significantly decreased from 47.77 to 29.36 (P < 0.001); and PC from 31.22 to 20.90 (P < 0.001). Also, the PDQ total scores significantly decreased from 79.00 to 50.26 (P < 0.001), suggesting a reduction in pain-related disability. Overall, surgery for degenerative cervical diseases improved the patients' physical health, reduced pain, lowered sadness, and dramatically reduced pain-related impairment.

**Table 2:** Analysis of 36-item Short Form Health Survey Quality of Life Scale (SF-36), Patient Health Questionnaire-9 (PHQ9), and The Pain Disability Questionnaire (PDQ) score before and after surgery.

		Before surgery	After surgery	P
SF-36	Physical functioning	57.57 ± 25.50	71.50 ± 21.09	0.003^a^
	Role-Physical	7.85 ± 20.80	50.00 ± 45.48	<0.001^a^
	Bodily pain	43.11 ± 16.38	61.90 ± 22.80	<0.001^a^
	General health	46.37 ± 20.68	57.86 ± 24.77	0.011^a^
	Vitality	42.85 ± 19.14	52.50 ± 19.81	<0.001^a^
	Social functioning	48.92 ± 25.43	64.16 ± 26.00	<0.001^a^
	Role-Emotional	31.42 ± 37.87	58.88 ± 43.49	0.010^a^
	Mental health	51.54 ± 20.32	60.53 ± 20.84	<0.001^a^
	Physical component score	38.72 ± 14.52	60.31 ± 23.98	<0.001^a^
	Mental component score	43.68 ± 20.99	59.02 ± 22.53	<0.001^a^
	Total	41.20 ± 16.29	59.66 ± 21.75	<0.001^a^
PHQ9		18.54 ± 5.28	15.00 ± 4.32	0.001^a^
PDQ	Functional Status Component	47.77 ± 19.29	29.36 ± 17.16	<0.001^a^
	Psychosocial Component	31.22 ± 13.24	20.90 ± 13.38	<0.001^a^
	Total	79.00 ± 30.60	50.26 ± 29.19	<0.001^a^

a indicated significant difference in the comparison of data between before and after surgery.

### 3.3 Holistic Well‐being scale (HWS) analysis before and after surgery

The HWS analysis in Table 3 demonstrated substantial changes in emotional vulnerability and the overall score of the affliction portion of spiritual well-being before and after surgery. Emotional vulnerability decreased significantly from 4.48 to 4.10 (P = 0.020), and the total HWS score also showed a significant improvement from 4.45 to 4.11 (P = 0.015). However, there were no significant changes in body irritability and spiritual disorientation in the affliction as well as in all items of equanimity.

**Table 3:** Holistic Well‐being Scale (HWS) analysis before and after surgery.

		Before surgery	After surgery	P
Affliction	Emotional vulnerability	4.48 ± 1.71	4.10 ± 1.60	0.020∗
	Bodily irritability	4.81 ± 2.12	4.30 ± 1.85	0.084
	Spiritual disorientation	3.97 ± 1.88	3.90 ± 1.66	0.785
	Total	4.45 ± 1.57	4.11 ± 1.39	0.015∗
Equanimity	Non-attachment	6.12 ± 1.95	6.46 ± 1.69	0.455
	Mindful awareness	6.86 ± 1.91	6.59 ± 2.03	0.187
	General vitality	5.95 ± 1.95	6.38 ± 1.90	0.328
	Spiritual self-care	6.09 ± 2.08	6.53 ± 2.16	0.462
	Total	6.26 ± 1.61	6.48 ± 1.70	0.734

Each item is rated using a ‘0 to 10’ scale, where ‘0'represents total disagreement and ‘10’ indicates total agreement. A high score of affliction indicates low spiritual resilience, whereas a high score of equanimity indicates high spiritual resilience. ∗indicated significant difference in the comparison of data between before and after surgery.

## 4 Discussion

The improvement in QOL observed in this study is in accordance with many studies publishing the impact of surgical intervention on patients’ QOL [[10], [11], [14], [15], [16], [17],^13^]. Fehlings et al. [10] evaluated the outcomes of surgical decompression for cervical myelopathy and found significant improvement in all domains of SF-36. Zhou et al. [15] found none of the domains of SF-36 showed statistically significant improvements at 3 months after surgery, while at 1 year after surgery, PF, RP, and SF showed significant improvements compared with preoperative scores. Al-Tomimi et al. [11] revealed that approximately 70 % of the patients experienced significant improvements in all outcome measures. About two-thirds of those patients who initially showed improvement maintained these significant improvements at the five-year follow-up. The systematic review [14] investigates the impact of surgical intervention on functional impairment, disability, and QOL in patients with DCM. The review reported significant improvements in Japanese Orthopaedic Association or modified scores, Neck Disability Index, and Visual Analogue Scale scores post-surgery, indicating a positive impact on functional status, disability, and pain and demonstrating the lasting benefits of surgical intervention. These studies support the effectiveness of surgical intervention in improving QOL, functional impairment, and pain in patients with degenerative cervical diseases [13].

This study revealed that the surgical treatment of degenerative cervical diseases not only addresses the physical aspects of the condition but also has a positive impact on the mental health of patients, as evidenced by reductions in depressive scores by PHQ9. The significant improvements in depressive symptoms post-surgery were also found by Parrish et al. [24] with the measurements by PHQ-9, 12-item Short Form Survey (SF-12), and MCS of Veterans RAND-12 scores. The strong correlations observed between SF-12 and MCS of Veterans RAND-12 scores with PHQ-9 scores both preoperatively and postoperatively indicate that PHQ-9 is a valid tool for evaluating depressive symptoms in this patient population. Similarly, He et al. [34] suggest that preoperative depressive symptoms, measured by PHQ-9, can predict postoperative outcomes in terms of pain and function. Lynch et al. [35] demonstrate that preoperative sleep difficulties, a component of depressive symptoms, predict clinically meaningful improvements in pain and function post-ACDF, underscoring the potential benefits of addressing sleep-related issues preoperatively. Notably, sex differences in the improvement of depressive symptoms post-surgery are noted by Cha et al. [36], indicating that while both sex show improvement, males may experience sustained benefits over a longer period. However, the sex differences were not analyzed in our study due to the limited sample size.

In this study, we discovered a significant improvement in the whole PDQ score, including functional and psychological components, in individuals with degenerative cervical diseases who underwent surgical intervention. It indicates the beneficial effects of surgical interventions on patient outcomes, such as pain control and disability reduction. Obviously, pain is a key determinant negatively influencing QOL in patients with DCM. A randomized controlled trial with long-term follow-up for around two decades focusing on pain, function, and mental factors demonstrated significant long-term improvements in neck pain and the Neck Disability Index [25], with 88 % of participants reporting improvements or full recovery, 71 % achieving a clinically relevant improvement in pain, and 41 % in disability. The severity and interference of pain were correlated with worse myelopathic severity and lower health-related QOL scores on the SF-36, indicating that pain in DCM is a multifaceted issue that severely affects individuals' daily living and well-being [1]. Overall, the benefits of surgical intervention for degenerative cervical diseases in terms of pain relief, disability, and QOL have been clearly demonstrated with consistent improvements [1,19,24,25,35].

While we observed significant improvements in emotional vulnerability and the overall affliction score of spiritual well-being according to the Holistic Well-being Scale (HWS) analysis, certain aspects—such as body irritability, spiritual disorientation within the affliction domain, and equanimity—showed no significant change post-surgery. These results indicate that, although surgery positively impacts some elements of spiritual well-being, the overall recovery of holistic well-being remains incomplete. This limited improvement in spiritual health may be attributed to the current limitations in traditional postoperative care, which typically focuses on physical rehabilitation and mental health but may not fully address spiritual needs. Additionally, spiritual well-being is often deeply connected to personal belief systems and support networks, which may not be directly influenced by surgery alone, suggesting that postoperative care must be extended to include specific spiritual interventions to support comprehensive recovery.

Spiritual care has been shown to reduce postoperative depression and anxiety, particularly in patients recovering from major surgeries like coronary artery bypass grafting surgery, where structured spiritual interventions, including prayer and meditation, significantly improve psychological outcomes by alleviating distress and fostering resilience [37,38]. For post-transplant patients, engaging in spiritual practices has been linked to better QOL and coping skills, enhancing emotional well-being and resilience through challenging recoveries [39]. To practically support spiritual health, healthcare providers may incorporate brief spiritual assessments and encourage practices like mindfulness, meditation, or community support, helping patients cultivate hope, connection, and purpose throughout recovery [40]. By adopting a more integrant approach that includes targeted support for mental and spiritual health alongside physical rehabilitation, healthcare teams may better address the multidimensional impacts of degenerative cervical disease, promoting a more complete and sustained recovery for patients.

The study underscores the effectiveness of surgical intervention in improving QOL, enhancing physical and mental health, reducing pain-related disability, and improving emotional vulnerability and total affliction among the patients. However, there are certain limitations to the investigation. First, the study sample was homogeneous, consisting of patients from a single medical center with similar socioeconomic and cultural backgrounds, primarily due to geographic location and convenience sampling, which limits the generalizability of the results to broader populations. Second, the six-month follow-up period restricts insights into the long-term effects of surgery, particularly on mental and spiritual health, which may evolve over time. Third, the study relied on subjective tools like the SF-36, PHQ-9, and HWS, which, though validated, are subject to personal bias due to individual interpretation and reporting styles. Furthermore, this observational study lacked randomization and did not control for comorbidities, limiting the ability to draw definitive conclusions about the effects of surgical intervention on degenerative cervical disorders. Finally, additional examination of subpopulation variables such as sex differences, preoperative symptoms such as cervical radiculopathy or DCM, and the patient's profile, was not feasible due to the restricted sample size. To mitigate these limitations, future research could focus on diversifying the sample population to enhance generalizability, extending the follow-up period to gain a better understanding of long-term effects, and incorporating both subjective and objective measures to provide a more comprehensive assessment of patient outcomes.

## 5 Conclusion

This study demonstrates that surgical interventions for degenerative cervical diseases significantly improve QOL, mental health, and pain relief, while highlighting the need for more attention to spiritual well-being. Clinicians, including spinal surgeons and rehabilitation specialists, can enhance postoperative care by incorporating mental health and spiritual support through brief assessments, referrals, and patient education on mindfulness practices. This holistic approach may improve overall recovery, satisfaction, and long-term QOL. This will better address the full spectrum of patient needs. Future research should focus on larger, more diverse populations, longer-term follow-up, and the incorporation of objective clinical measures alongside patient-reported outcomes. Additionally, studies should explore targeted interventions aimed at improving spiritual health to ensure comprehensive care for these patients.

## CRediT authorship contribution statement

**Ching-Ya Huang:** Writing – original draft. **Cheng-Shyuan Rau:** Writing – review & editing, Funding acquisition. **Jo-Chien Lin:** Resources. **Shiun-Yuan Hsu:** Formal analysis. **Ching-Hua Hsieh:** Supervision, Conceptualization.

## Ethics statement

Prior to implementation, this study was approved by the hospital's Institutional Review Board under protocol number 202200381B0, and patient consent was obtained. This report adheres to the principles outlined in Elsevier's Publishing Ethics Policy.

## Data availability statement

The de-identification data could only be provided for academic research via the corresponding author, under the permission of Institutional Review Board.

## Funding

This research was funded by 10.13039/100012553Chang Gung Memorial Hospital, grant number CORPG8M0301 to Cheng-Shyuan Rau.

## Declaration of competing interest

The authors declare the following financial interests/personal relationships which may be considered as potential competing interests:Cheng-Shyuan Rau reports a relationship with 10.13039/501100011892Kaohsiung Chang Gung Memorial Hospital that includes: funding grants. If there are other authors, they declare that they have no known competing financial interests or personal relationships that could have appeared to influence the work reported in this paper.
