# Supplementary material for: Surgical interventions for degenerative cervical disease: Impact on patient quality of life, mental health, pain relief, and spiritual health
Source: Heliyon. 2024 Dec 27;11(1):e41555. doi: 10.1016/j.heliyon.2024.e41555 (PMC11755049; doi:10.1016/j.heliyon.2024.e41555)
Supplement: Multimedia component 1 [file mmc1.pdf]

Questionnaire I: Basic Personal Information

Please check ✓ for the answer that matches your answer. Thank you very much for your cooperation!

Study number:

Time point: Before surgery Fill in date: _____date____Month____Year

1. Age: Born in January of the year of the Republic of China.
2. Gender: □1. male □2. female
3. Height: _____cm ; Weight:_____kg
4. Marital Status: □1. Unmarried □2. Married □3. Divorced □4. Separated □5. Cohabiting □6. Widowed
5. Occupation: □1. No □2. Housekeeping □3. worker □4. merchant □5. public servant □6. Others_____
6. Education level: □1. Illiterate □2. Elementary school □3. Junior high school □4. High school □5. College □ 6. University □ 7. Master Doctorate (or above)
7. Religion: □1. None □2. Buddhism □3. Taoism □4. Christianity □5. Catholicism □6. Yilan Tao □7. Muslims □8. Other_____
8. Chronic Diseases: □1. Diabetes □2. Hypertension □3. Heart disease □4. History of stroke □5. Renal insufficiency □6. Hyperlipidemia □7. Hepatitis □8. Others_____
9. Smoking: □No □Yes; Drinking: □No □Yes; Betel nut: □No chewing □Chewing
10. Hospitalized primary companion (check all that apply):
    □1. spouse or cohabitant □2. parents □3. siblings □4. children □5. relatives
    □6. neighbors □7. friends □8. Religious or social group activities □9. Social welfare organization □10. Other_____
11. Estimated primary companion after discharge from the hospital (check all that apply):
    □1. spouse or cohabitant □2. parents □3. siblings □4. Children □5. relatives
    □6. neighbors □7. Friends □8. Religious or social group activities □9. Social welfare organizations □10. Other_____
