# Supplementary material for: Surgical interventions for degenerative cervical disease: Impact on patient quality of life, mental health, pain relief, and spiritual health
Source: Heliyon. 2024 Dec 27;11(1):e41555. doi: 10.1016/j.heliyon.2024.e41555 (PMC11755049; doi:10.1016/j.heliyon.2024.e41555)
Supplement: Multimedia component 2 [file mmc2.pdf]

Appendix A. Pain Dysfunction Questionnaire

1) **Does your pain interfere with your normal work inside and outside**
NAME:______________________ DATE:__________

Please read:
This survey asks for your views about how your pain now affects how you function in everyday activities. This information will help you and your doctor know how you feel and how well you are able to do your daily tasks at this time.

Please answer every question by making an "X" along the line to show how much your pain problem has affected you (from having no problems at all to having the most severe problems you can imagine).

BE SURE TO ANSWER ALL QUESTIONS.

1) **Does your pain interfere with your normal work inside and outside the home?**
]__________]__________]__________]__________]__________]
Work normally — Unable to work at all

2) **Does your pain interfere with personal care (such as washing, dressing, etc.)?**
]__________]__________]__________]__________]__________]
Take care of myself completely — Need help with all my personal care

3) **Does your pain interfere with your traveling?**
]__________]__________]__________]__________]__________]
Travel anywhere I like — Only travel to see doctors

4) **Does your pain affect your ability to sit or stand?**
]__________]__________]__________]__________]__________]
No problems — Cannot sit/stand at all

5) **Does your pain affect your ability to lift overhead, grasp objects, or reach for things?**
]__________]__________]__________]__________]__________]
No problems — Cannot do at all

6) **Does your pain affect your ability to lift objects off the floor, bend, stoop, or squat?**
]__________]__________]__________]__________]__________]
No problems — Cannot do at all

7) **Does your pain affect your ability to walk or run?**
]__________]__________]__________]__________]__________]
No problems — Cannot walk/run at all

8) **Has your income declined since your pain began?**
]__________]__________]__________]__________]__________]
No decline — Lost all income

9) **Do you have to take pain medication every day to control your pain?**
]__________]__________]__________]__________]__________]
No medication needed — On pain medication throughout the day

10) **Does your pain force you to see doctors much more often than before your pain began?**
]__________]__________]__________]__________]__________]
Never see doctors — See doctors weekly

11) **Does your pain interfere with your ability to see the people who are important to you as much as you would like?**
]__________]__________]__________]__________]__________]
No problem — Never see them

12) **Does your pain interfere with recreational activities and hobbies that are important to you?**
]__________]__________]__________]__________]__________]
No interference — Total interference

13) **Do you need the help of your family and friends to complete everyday tasks (including both work outside the home and housework) because of your pain?**
]__________]__________]__________]__________]__________]
Never need help — Need help all the time

14) **Do you now feel more depressed, tense, or anxious than before your pain began?**
]__________]__________]__________]__________]__________]
No depression/tension — Severe depression/tension

15) **Are there emotional problems caused by your pain that interfere with your family, social, or work activities?**
]__________]__________]__________]__________]__________]
No problems — Severe problems

Copyright © Lippincott Williams & Wilkins. Unauthorized reproduction of this article is prohibited.

Appendix B. Pain Dysfunction Questionnaire Scoring

Every 1.5 cm = 1 increment

0	1	2	3	4	5	6	7	8	9	10

]__________]__________]__________]__________]__________]

If an “X” is exactly on the line between one number and the next, then it is scored as the lower number. If the “X” is one “millimicron” over into the next increment, then score “up.” Score a value for each line, and sum the total for all 15 lines. If the patient has put 2 “X’s” on the line, use the point that is halfway between the two points as the item score. For example, if an “X” is marked at 2 and an “X” at 6, one would score this particular item a 4.

The PDQ is made up of two factors: a Functional Status Component and a Psychosocial Component. To differentiate these two components, one must separate the scores.

A) Functional Status Component: Total the scores for items 1, 2, 3, 4, 5, 6, 7, 12, and 13 (maximum = 90).
B) Psychosocial Component: Total the scores for items 8, 9, 10, 11, 14, and 15 (maximum = 60).
C) Total PDQ Score: Total of the scores of all items (should equal to Functional Status Score + Psychosocial Component Score).

Blank Items: If some lines are left blank, they should be pro-rated. To do this, one must first determine whether the item is part of the Functional Status or Psychosocial Component. Then, one would calculate the total component score and divide by the number of component items answered to obtain a mean. This mean score would then be added to each item left blank for that particular component. For example, if a patient leaves question 5 blank, one would calculate the total for the Functional Status Component. Suppose that the 8 items answered sum to 48. One would then divide 48 by the number of items answered. In this case, 8 were answered, so the mean item score for the Functional Status Component is 6. One would then add 6 to the Functional Status Component, which for this example would be 54. When computing the total PDQ score, this of course adds 6 points as well.

The same is true for the Psychosocial Component, although one must be careful because there are only 6 items comprising this component. For example, if the same patient mentioned above also leaves question 14 blank, one would have to pro-rate this item for the Psychosocial Component. If the remaining 5 questions for the Psychosocial Component are answered and sum to 30, we would have a mean item score of 6 for the Psychosocial Component. Again, one would add 6 points to the Psychosocial Component Total. Now, the Psychosocial Component will equal 36, and the total of the Functional Status and Psychosocial Components equals a total PDQ score of 90.

An example is provided on the following page. After scoring this particular PDQ, this is how the total and component scores would read:

Total PDQ Score = 67

Functional Status Component = 41

Psychosocial Component = 26

Copyright © Lippincott Williams & Wilkins. Unauthorized reproduction of this article is prohibited.
