# Supplementary material for: Surgical interventions for degenerative cervical disease: Impact on patient quality of life, mental health, pain relief, and spiritual health
Source: Heliyon. 2024 Dec 27;11(1):e41555. doi: 10.1016/j.heliyon.2024.e41555 (PMC11755049; doi:10.1016/j.heliyon.2024.e41555)
Supplement: Multimedia component 4 [file mmc4.pdf]

Questionnaire IV: Taiwan version of SF-36 Health Scale

Study Number:

Time point: □ Before surgery □ Six months after surgery

Fill in date: ____date____Month____Year

The purpose of this survey is to explore your perception of your health. This information will help you keep track of how you feel and how well you are able to perform your daily activities.

Please answer the following questions and circle the appropriate answer. If you are not sure about your answer to a question, please choose the most appropriate answer possible. For the purpose of this section, the past month means the first 30 days from today

Choose one option for each questionnaire item.

1. In general, would you say your health is:

○ 1 - Excellent

○ 2 - Very good

○ 3 - Good

○ 4 - Fair

○ 5 - Poor

---

2. **Compared to one year ago**, how would you rate your health in general **now**?

○ 1 - Much better now than one year ago

○ 2 - Somewhat better now than one year ago

○ 3 - About the same

○ 4 - Somewhat worse now than one year ago

○ 5 - Much worse now than one year ago

The following items are about activities you might do during a typical day. Does **your health now limit you** in these activities? If so, how much?

	Yes, limited a lot	Yes, limited a little	No, not limited at all
3. **Vigorous activities**, such as running, lifting heavy objects, participating in strenuous sports	○ 1	○ 2	○ 3
4. **Moderate activities**, such as moving a table, pushing a vacuum cleaner, bowling, or playing golf	○ 1	○ 2	○ 3
5. Lifting or carrying groceries	○ 1	○ 2	○ 3
6. Climbing **several** flights of stairs	○ 1	○ 2	○ 3
7. Climbing **one** flight of stairs	○ 1	○ 2	○ 3
8. Bending, kneeling, or stooping	○ 1	○ 2	○ 3
9. Walking **more than a mile**	○ 1	○ 2	○ 3
10. Walking **several blocks**	○ 1	○ 2	○ 3
11. Walking **one block**	○ 1	○ 2	○ 3
12. Bathing or dressing yourself	○ 1	○ 2	○ 3

During the **past 4 weeks**, have you had any of the following problems with your work or other regular daily activities **as a result of your physical health**?

	Yes	No
13. Cut down the **amount of time** you spent on work or other activities	◯ 1	◯ 2
14. **Accomplished less** than you would like	◯ 1	◯ 2
15. Were limited in the **kind** of work or other activities	◯ 1	◯ 2
16. Had **difficulty** performing the work or other activities (for example, it took extra effort)	◯ 1	◯ 2

During the **past 4 weeks**, have you had any of the following problems with your work or other regular daily activities **as a result of any emotional problems** (such as feeling depressed or anxious)?

	Yes	No
17. Cut down the **amount of time** you spent on work or other activities	◯ 1	◯ 2
18. **Accomplished less** than you would like	◯ 1	◯ 2
19. Didn't do work or other activities as **carefully** as usual	◯ 1	◯ 2

20. During the **past 4 weeks**, to what extent has your physical health or emotional problems interfered with your normal social activities with family, friends, neighbors, or groups?

◯ 1 - Not at all

◯ 2 - Slightly

◯ 3 - Moderately

- 4 - Quite a bit
- 5 - Extremely

21. How much **bodily** pain have you had during the **past 4 weeks**?

- 1 - None
- 2 - Very mild
- 3 - Mild
- 4 - Moderate
- 5 - Severe
- 6 - Very severe

22. During the **past 4 weeks**, how much did **pain** interfere with your normal work (including both work outside the home and housework)?

- 1 - Not at all
- 2 - A little bit
- 3 - Moderately
- 4 - Quite a bit
- 5 - Extremely

These questions are about how you feel and how things have been with you **during the past 4 weeks**. For each question, please give the one answer that comes closest to the way you have been feeling.

How much of the time during the **past 4 weeks**...

	All of the time	Most of the time	A good bit of the time	Some of the time	A little of the time	None of the time
23. Did you feel full of pep?	◯ 1	◯ 2	◯ 3	◯ 4	◯ 5	◯ 6
24. Have you been a very nervous person?	◯ 1	◯ 2	◯ 3	◯ 4	◯ 5	◯ 6
25. Have you felt so down in the dumps that nothing could cheer you up?	◯ 1	◯ 2	◯ 3	◯ 4	◯ 5	◯ 6
26. Have you felt calm and peaceful?	◯ 1	◯ 2	◯ 3	◯ 4	◯ 5	◯ 6
27. Did you have a lot of energy?	◯ 1	◯ 2	◯ 3	◯ 4	◯ 5	◯ 6
28. Have you felt downhearted and blue?	◯ 1	◯ 2	◯ 3	◯ 4	◯ 5	◯ 6
29. Did you feel worn out?	◯ 1	◯ 2	◯ 3	◯ 4	◯ 5	◯ 6
30. Have you been a happy person?	◯ 1	◯ 2	◯ 3	◯ 4	◯ 5	◯ 6
31. Did you feel tired?	◯ 1	◯ 2	◯ 3	◯ 4	◯ 5	◯ 6

---

32. During the **past 4 weeks**, how much of the time has **your physical health or emotional problems** interfered with your social activities (like visiting with friends, relatives, etc.)?

◯ 1 - All of the time

◯ 2 - Most of the time

◯ 3 - Some of the time

- 4 - A little of the time
- 5 - None of the time

How TRUE or FALSE is **each** of the following statements for you.

	Definitely true	Mostly true	Don't know	Mostly false	Definitely false
33. I seem to get sick a little easier than other people	◯ 1	◯ 2	◯ 3	◯ 4	◯ 5
34. I am as healthy as anybody I know	◯ 1	◯ 2	◯ 3	◯ 4	◯ 5
35. I expect my health to get worse	◯ 1	◯ 2	◯ 3	◯ 4	◯ 5
36. My health is excellent	◯ 1	◯ 2	◯ 3	◯ 4	◯ 5
