# Supplementary material for: Surgical interventions for degenerative cervical disease: Impact on patient quality of life, mental health, pain relief, and spiritual health
Source: Heliyon. 2024 Dec 27;11(1):e41555. doi: 10.1016/j.heliyon.2024.e41555 (PMC11755049; doi:10.1016/j.heliyon.2024.e41555)
Supplement: Multimedia component 5 [file mmc5.pdf]

Body-Mind-Spirit Holistic Well-being Scale

Please choose the response which best describes you at this point of time for each of the following statements.

In general...	Totally disagree									Totally agree
1. I mind about the negative views people have about me.	1	2	3	4	5	6	7	8	9	10
2. I feel hopeless.	1	2	3	4	5	6	7	8	9	10
3. My feelings are easily hurt.	1	2	3	4	5	6	7	8	9	10
4. I am a valuable person.	1	2	3	4	5	6	7	8	9	10
5. I am at peace with whatever life holds in store for me.	1	2	3	4	5	6	7	8	9	10
6. I tend to hold grudges and find it hard to forgive those who've hurt me.	1	2	3	4	5	6	7	8	9	10
7. I am in a bad mood when I wake up in the morning.	1	2	3	4	5	6	7	8	9	10
8. I feel restless and uneasy.	1	2	3	4	5	6	7	8	9	10
9. I often seek inner peace.	1	2	3	4	5	6	7	8	9	10
10. I am enthusiastic about my life.	1	2	3	4	5	6	7	8	9	10
11. I am aware of the needs of others.	1	2	3	4	5	6	7	8	9	10
12. I can accept changes in life with a sense of ease.	1	2	3	4	5	6	7	8	9	10
13. I am able to be aware of both my physical condition and bodily sensations.	1	2	3	4	5	6	7	8	9	10
14. I can fully concentrate on what I am doing.	1	2	3	4	5	6	7	8	9	10
15. I am full of energy.	1	2	3	4	5	6	7	8	9	10
16. I have a rich religious / spiritual life.	1	2	3	4	5	6	7	8	9	10
17. I feel like my head is throbbing.	1	2	3	4	5	6	7	8	9	10
18. I can take ups and downs in life as they come.	1	2	3	4	5	6	7	8	9	10
19. I can pick up changes of others' emotional states.	1	2	3	4	5	6	7	8	9	10
20. My whole life seems to be meaningless.	1	2	3	4	5	6	7	8	9	10
21. If others treat me unfairly, I'd feel upset for a long time.	1	2	3	4	5	6	7	8	9	10
22. I am able to pick up changes in my mood.	1	2	3	4	5	6	7	8	9	10
23. I sleep well.	1	2	3	4	5	6	7	8	9	10
24. I can let go if I so desire.	1	2	3	4	5	6	7	8	9	10
25. I am able to accept the many regrets in my life.	1	2	3	4	5	6	7	8	9	10
26. I have lost my direction in life.	1	2	3	4	5	6	7	8	9	10
27. I can take care of both the needs of my mind and body.	1	2	3	4	5	6	7	8	9	10
28. I cannot let go of many things in my life.	1	2	3	4	5	6	7	8	9	10
29. I feel irritable.	1	2	3	4	5	6	7	8	9	10
30. My body is very tense and taut.	1	2	3	4	5	6	7	8	9	10
