# Supplementary material for: Surgical interventions for degenerative cervical disease: Impact on patient quality of life, mental health, pain relief, and spiritual health
Source: Heliyon. 2024 Dec 27;11(1):e41555. doi: 10.1016/j.heliyon.2024.e41555 (PMC11755049; doi:10.1016/j.heliyon.2024.e41555)
Supplement: Multimedia component 6 [file mmc6.docx]

**問卷一、個人基本資料**

請在符合您的答案項目□內打✓，非常感謝您的合作

研究編號：

填寫時間點: 手術前 填寫日期: 年 月 日

1.年齡：­­民國 ­年 月出生

2. 性別：□1.男 □2.女

3. 身高: 公分; 體重： 公斤

4. 婚姻狀況：□1.未婚□2.已婚□3.離婚□4.分居□5.同居□6.喪偶

5. 職業狀況：：□1.無□2.家管□3.工□4.商□5.公□6.其他

6. 教育程度：□1.不識字□2.國小□3.國中□4.高中□5.專科□6.大學□7.碩士 博士(含)以上

7. 宗教信仰：□1.無□2.佛教□3.道教□4.基督教□5.天主教 □6.一貫道 □7.回教

□8. 其它

8. 慢性疾病：□1.糖尿病□2.高血壓□3.心臟病□4.中風史□5.腎臟功能不全 □6.高

血脂 □7.肝炎 □8.其它

9. **抽菸**：□無 □有；**喝酒**： □無 □有；**檳榔**：□無嚼食 □有嚼食

10. 住院中主要陪伴者(可複選)：

□1.配偶或同居人□2. 父母□3. 兄弟姊妹□4. 子女 5. 親戚□6. 鄰居 □7.朋友

□8. 宗教或社會團體活動 □9.社福機構 □10.其他

11. 出院後預計主要陪伴者(可複選)：

□1.配偶或同居人□2. 父母□3. 兄弟姊妹□4. 子女 5. 親戚□6. 鄰居 □7.朋友

□8. 宗教或社會團體活動 □9.社福機構 □10.其他
