# Supplementary material for: Surgical interventions for degenerative cervical disease: Impact on patient quality of life, mental health, pain relief, and spiritual health
Source: Heliyon. 2024 Dec 27;11(1):e41555. doi: 10.1016/j.heliyon.2024.e41555 (PMC11755049; doi:10.1016/j.heliyon.2024.e41555)
Supplement: Multimedia component 7 [file mmc7.docx]

**問卷二、疼痛失能問卷(PDQ)**

研究編號：

填寫時間: □手術前 □手術後半年 填寫日期: 年 月 日

請閱讀：本調查詢問您對您的疼痛如何影響您在日常活動中的運作方式的看法。這信息將幫助您和您的醫生了解您的感受以及此時您完成日常任務的能力。

請你在每一個問題上畫一個“✓”，以顯示您的疼痛問題對您的影響有多大

(從完全沒有問題1分到遇到你能想像到的最嚴重的問題10分)

|  | 1 | 2 | 3 | 4 | 5 | 6 | 7 | 8 | 9 | 10 |
| --- | --- | --- | --- | --- | --- | --- | --- | --- | --- | --- |
| 1. 您的疼痛是否會干擾您在家內外的正常工作嗎？ |  |  |  |  |  |  |  |  |  |  |
| 1. 您的疼痛是否會影響個人護理(如洗滌、穿衣等)嗎？ |  |  |  |  |  |  |  |  |  |  |
| 1. 你的疼痛會影響你的旅行嗎？ |  |  |  |  |  |  |  |  |  |  |
| 1. 您的疼痛是否會影響您坐下或站立的能力嗎? |  |  |  |  |  |  |  |  |  |  |
| 1. 您的疼痛是否會影響您抬過頭頂、抓握物體或伸手去拿東西的能力嗎？ |  |  |  |  |  |  |  |  |  |  |
| 1. 您的疼痛是否會影響您將物體抬離地板、彎曲、彎腰或蹲下的能力？ |  |  |  |  |  |  |  |  |  |  |
| 1. 你的疼痛會影響你走路或跑步的能力嗎? |  |  |  |  |  |  |  |  |  |  |
| 1. 自從你開始疼痛以來，你的收入有沒有下降？ |  |  |  |  |  |  |  |  |  |  |
| 1. 你必須每天服用止痛藥來控制你的疼痛嗎？ |  |  |  |  |  |  |  |  |  |  |
| 1. 您的疼痛是否迫使您比疼痛開始前更頻繁地去看醫生嗎？ |  |  |  |  |  |  |  |  |  |  |
| 1. 您的疼痛是否會干擾您隨心所欲地看到對您很重要的人的能力？ |  |  |  |  |  |  |  |  |  |  |
| 1. 您的疼痛是否會干擾對您很重要的娛樂活動和愛好嗎？ |  |  |  |  |  |  |  |  |  |  |
| 1. 您是否因為疼痛而需要家人和朋友的幫助才能完成日常任務(包括外出工作和家務)？ |  |  |  |  |  |  |  |  |  |  |
| 1. 您現在比疼痛開始前感到更加沮喪、緊張或焦慮嗎？ |  |  |  |  |  |  |  |  |  |  |
| 1. 您的疼痛是否會導致影響您的家庭、社交或工作活動的情緒問題？ |  |  |  |  |  |  |  |  |  |  |
