# Supplementary material for: Surgical interventions for degenerative cervical disease: Impact on patient quality of life, mental health, pain relief, and spiritual health
Source: Heliyon. 2024 Dec 27;11(1):e41555. doi: 10.1016/j.heliyon.2024.e41555 (PMC11755049; doi:10.1016/j.heliyon.2024.e41555)
Supplement: Multimedia component 8 [file mmc8.docx]

**問卷三、病人健康問卷(PHQ-9)**

在過去兩個星期， 有多少時候您受到以下任何問題所困擾？請勾選(✓)最適合的答案

研究編號：

填寫時間: □手術前 □手術後半年 填寫日期: 年 月 日

1. **作任何事都覺得沉悶或者根本不想做任何事**

|  | 完全沒有 |
| --- | --- |
|  | 有幾天 |
|  | 一半以上的天數 |
|  | 幾乎每天 |

1. **情緒低落、抑鬱或絕望**

|  | 完全沒有 |
| --- | --- |
|  | 有幾天 |
|  | 一半以上的天數 |
|  | 幾乎每天 |

1. **難於入睡；半夜會醒或相反地睡覺時間過多**

|  | 完全沒有 |
| --- | --- |
|  | 有幾天 |
|  | 一半以上的天數 |
|  | 幾乎每天 |

1. **覺得疲倦或活力不足**

|  | 完全沒有 |
| --- | --- |
|  | 有幾天 |
|  | 一半以上的天數 |
|  | 幾乎每天 |

1. **胃口極差或進食過量**

|  | 完全沒有 |
| --- | --- |
|  | 有幾天 |
|  | 一半以上的天數 |
|  | 幾乎每天 |

1. **不喜歡自己，覺得自己做的不好、對自己失望或有負家人的期望**

|  | 完全沒有 |
| --- | --- |
|  | 有幾天 |
|  | 一半以上的天數 |
|  | 幾乎每天 |

1. **難於集中精神做事，例如看報紙或看電視**

|  | 完全沒有 |
| --- | --- |
|  | 有幾天 |
|  | 一半以上的天數 |
|  | 幾乎每天 |

1. **其他人反應你行動或說話遲緩；或者相反地，你比平常活動更多，坐立不安、停不下來**

|  | 完全沒有 |
| --- | --- |
|  | 有幾天 |
|  | 一半以上的天數 |
|  | 幾乎每天 |

1. **想到自己最好去死或自殘**

|  | 完全沒有 |
| --- | --- |
|  | 有幾天 |
|  | 一半以上的天數 |
|  | 幾乎每天 |
