# Supplementary material for: Surgical interventions for degenerative cervical disease: Impact on patient quality of life, mental health, pain relief, and spiritual health
Source: Heliyon. 2024 Dec 27;11(1):e41555. doi: 10.1016/j.heliyon.2024.e41555 (PMC11755049; doi:10.1016/j.heliyon.2024.e41555)
Supplement: Multimedia component 10 [file mmc10.doc]

**問卷四、臺灣SF-36健康量表**

研究編號：

填寫時間: □手術前 □手術後半年 填寫日期: 年 月 日

本調查目的在探討您對自己健康的看法。這些資訊將能幫助您記錄您的感受，以及您在執行日常生活的能力。

敬請回答下列各問題並圈選一適當答案。如您對某一問題的回答不能確定，還是請您盡可能選一個最適合的答案。在本部份所指過去一個月內，係指從今天往前算三十天內。

1. 一般來說，您認為您目前的健康狀況是（請僅圈選一項答案）

極好的................................................................................................................1

很好....................................................................................................................2

好.........................................................................................................................3

普通....................................................................................................................4

不好....................................................................................................................5

2. 和一年前比較，您認為您目前的健康狀況是？（請僅圈選一項答案）

比一年前好很多................................................................................................1

比一年前好一些................................................................................................2

和一年前差不多................................................................................................3

比一年前差一些................................................................................................4

比一年前差很多................................................................................................5

3. 下面是一些您日常可能從事的活動，請問您目前健康狀況會不會限制您從事這些

活動？如果會，到底限制有多少？ （每行請僅圈選一項答案）

| **活 動** | **會,**  **受到很多限制** | **會,**  **受到一些限制** | **不會,**  **完全不受限制** |
| --- | --- | --- | --- |
| a.**費力活動**，例如跑步、提重物、  參與劇烈運動 |  |  |  |
| b.**中等程度活動**，例如搬桌子、  拖地板、打保齡球、或打太極  拳 |  |  |  |
| c.提起或攜帶食品雜貨 |  |  |  |
| d.爬**數**層樓樓梯 |  |  |  |
| e.爬**一**層樓樓梯 |  |  |  |
| f.彎腰、跪下或蹲下 |  |  |  |
| g.走路**超過1公里** |  |  |  |
| h.走過**數個街口** |  |  |  |
| i.走過**一個街口** |  |  |  |
| j.自己洗澡或穿衣 |  |  |  |

4.在過去一個月內，您是否曾因為身體健康問題，而在工作上或其他日常活動方 面有下列任何的問題?（每行請僅圈選一項答案）

|  | **是** | **否** |
| --- | --- | --- |
| a.做工作或其它活動的**時間**減少 |  |  |
| b.完成的工作量比您想要**完成的較少** |  |  |
| c.可以做的工作或其他活動的**種類**受到限制 |  |  |
| d.做工作或其他活動**有困難** (例如，須更吃力) |  |  |

5.在過去一個月內，您是否曾因為情緒問題(例如，感覺沮喪或焦慮)，而在工作 上或其他日常活動方面有下列的問題? (每行請僅圈選一項答案）

|  | **是** | **否** |
| --- | --- | --- |
| a.做工作或其它活動的**時間**減少 |  |  |
| b.完成的工作量比您想要**完成的較少** |  |  |
| c.做工作或其它活動時不如以往**小心** |  |  |

6. 在過去一個月內，您的身體健康或情緒問題，對您與家人或朋友、鄰居、社團間的平常

活動的妨礙程度如何？ (請僅圈選一項答案)

完全沒有妨礙........................................................................................................1

有一點妨礙........................................................................................................2

中度妨礙. ........................................................................................................3

相當多妨礙........................................................................................................4

妨礙到極點........................................................................................................5

7. 在過去一個月內，您身體疼痛程度有多嚴重？ (請僅圈選一項答案)

完全不痛............................................................................................................1

非常輕微的痛............................................................................................................2

輕微的痛............................................................................................................3

中度的痛............................................................................................................4

嚴重的痛............................................................................................................5

非常嚴重的痛

............................................................................................................6

8. 在過去一個月內，身體疼痛對您的日常工作(包括上班及家務)妨礙程度如何？(請僅圈選一項答案)

完全沒有妨礙............................................................................................................1

有一點妨礙............................................................................................................2

中度妨礙............................................................................................................3

相當多妨礙............................................................................................................4

妨礙到極點............................................................................................................5

9. 下列各項問題是關於過去一個月內您的感覺及您對周遭生活的感受，請針對每一問題選一最接近您感覺的答案。在過去一個月中有多少時候......（每行請僅圈選一項答案）

|  | **一直都是** | **大部分時間** | **經常** | **有時** | **很少** | **從不** |
| --- | --- | --- | --- | --- | --- | --- |
| a.您覺得充滿活力？ |  |  |  |  |  |  |
| b.您是一個非常緊張的人？ |  |  |  |  |  |  |
| c.您覺得非常沮喪，沒有任何事情  可以讓您高興起來？ |  |  |  |  |  |  |
| d.您覺得心情平靜？ |  |  |  |  |  |  |
| e.您精力充沛？ |  |  |  |  |  |  |
| f.您覺得悶悶不樂和憂鬱？ |  |  |  |  |  |  |
| g.您覺得筋疲力竭？ |  |  |  |  |  |  |
| h.您是一個快樂的人？ |  |  |  |  |  |  |
| i.您覺得累？ |  |  |  |  |  |  |

10. 在過去一個月內，您的身體健康或情緒問題有多少時候會妨礙您的社交活動（如拜訪親友等）？

(請僅圈選一項答案)

一直都會.....................................................................................................................1

大部分時間會.....................................................................................................................2

有時候會.....................................................................................................................3

很少會.....................................................................................................................4

從不會.....................................................................................................................5

11.下列各個陳述對您來說有多正確？

(每行請僅圈選一項答案)

|  | **完全正確** | **大部分正確** | **不知道** | **大部分不正確** | **完全**  **不正確** |
| --- | --- | --- | --- | --- | --- |
| a.我好像比別人較容易生病 |  |  |  |  |  |
| b.和任何一個我認識的人來比,我和他們一樣健康。 |  |  |  |  |  |
| c.我想我的健康會越來越壞 |  |  |  |  |  |
| d.我的健康狀況好得很 |  |  |  |  |  |
